# Development of a Digital Health Intervention for the Secondary Prevention of Cardiovascular Disease (INTERCEPT): Co-Design and Usability Testing Study

**DOI:** 10.2196/63707

**Published:** 2024-10-23

**Authors:** Irene Gibson, Lis Neubeck, Marissa Corcoran, Chris Morland, Steve Donovan, Jennifer Jones, Caroline Costello, Lisa Hynes, Aisling Harris, Mary Harrahill, Mary Lillis, Alison Atrey, Chantal F Ski, Vilius Savickas, Molly Byrne, Andrew W Murphy, John William McEvoy, David Wood, Catriona Jennings

**Affiliations:** 1 School of Medicine University of Galway Galway Ireland; 2 National Institute for Prevention and Cardiovascular Health Galway Ireland; 3 School of Health and Social Care Edinburgh Napier University Edinburgh United Kingdom; 4 St James’s Hospital Dublin Ireland; 5 Citrus Suite Liverpool United Kingdom; 6 Croí, West of Ireland Cardiac and Stroke Foundation Galway Ireland; 7 Public and Patient Involvement Panel Croí, West of Ireland Cardiac and Stroke Foundation Galway Ireland; 8 School of Nursing and Midwifery Queen’s University Belfast Belfast United Kingdom; 9 School of Chemistry, Pharmacy and Pharmacology University of East Anglia Norwich United Kingdom; 10 Health Behaviour Change Research Group University of Galway Galway Ireland; 11 Health Research Board Primary Care Clinical Trials Network Ireland University of Galway Galway Ireland

**Keywords:** cardiovascular disease, secondary prevention, digital health, intervention development, co-design, usability testing, mobile health, usability, design, conline workshop, social support, behavioral change, self-monitoring

## Abstract

**Background:**

Secondary prevention is an important strategy to reduce the burden of cardiovascular disease (CVD), a leading cause of death worldwide. Despite the growing evidence for the effectiveness of digital health interventions (DHIs) for the secondary prevention of CVD, the majority are designed with minimal input from target end users, resulting in poor uptake and usage.

**Objective:**

This study aimed to optimize the acceptance and effectiveness of a DHI for the secondary prevention of CVD through co-design, integrating end users’ perspectives throughout.

**Methods:**

A theory-driven, person-based approach using co-design was adopted for the development of the DHI, known as INTERCEPT. This involved a 4-phase iterative process using online workshops. In phase 1, a stakeholder team of health care professionals, software developers, and public and patient involvement members was established. Phase 2 involved identification of the guiding principles, content, and design features of the DHI. In phase 3, DHI prototypes were reviewed for clarity of language, ease of navigation, and functionality. To anticipate and interpret DHI usage, phase 4 involved usability testing with participants who had a recent cardiac event (<2 years). To assess the potential impact of usability testing, the System Usability Scale was administered before and after testing. The GUIDED (Guidance for Reporting Intervention Development Studies in Health Research) checklist was used to report the development process.

**Results:**

Five key design principles were identified: simplicity and ease of use, behavioral change through goal setting and self-monitoring, personalization, system credibility, and social support. Usability testing resulted in 64 recommendations for the app, of which 51 were implemented. Improvements in System Usability Scale scores were observed when comparing the results before and after implementing the recommendations (61 vs 83; *P*=.02).

**Conclusions:**

Combining behavior change theory with a person-based, co-design approach facilitated the development of a DHI for the secondary prevention of CVD that optimized responsiveness to end users’ needs and preferences, thereby potentially improving future engagement.

## Introduction

Patients with coronary heart disease (CHD) are at high risk of recurrent cardiovascular events [1], with 1 in 5 experiencing a recurrent event in the first year after hospital discharge [2]. To minimize this risk, guidelines recommend early initiation of evidence-based, secondary prevention lifestyle and pharmacological treatments, together with access to a cardiac rehabilitation (CR) program [1,3]. However, in reality, only a minority of patients with CHD achieve optimal risk factor control and less than a half attend CR, usually several weeks or even months after hospital discharge [4]. The promising role of digital health interventions (DHIs), including mobile health (mHealth) apps, in addressing this implementation gap is increasingly recognized [5]. Indeed, the widespread availability of smartphones and mobile devices means that mHealth apps are one of the most common types of DHIs being developed to improve the secondary prevention of CHD [6,7].

mHealth apps have the potential to enhance patient empowerment and self-management [8], with growing evidence to support their use for secondary prevention of cardiovascular disease (CVD) [5]. Recent meta-analysis data show that mHealth apps can improve exercise capacity, physical activity, adherence to medication, and quality of life, as well as reduce hospital readmissions in patients with CHD [9]. However, beyond the research setting, the uptake and usage of health apps is low [6,10,11]. In addition, there is limited understanding of how these apps are developed and what their “active ingredients” include [12,13].

Involving end users in the design and development of DHIs is recognized as essential to maximizing their acceptance, uptake, and effectiveness [5,14]. Despite this, the majority of mHealth apps are designed with minimal input from target end users [13]. For example, in a recent scoping review, the use of co-design methodologies in the development of CVD secondary prevention interventions was reported in only 4 out of 22 studies related to mHealth apps [15]. Furthermore, none of these studies reported using a theoretical framework to guide the development of the intervention, including the use of theory-based behavioral strategies. Although this absence of reporting is not uncommon [16], it impacts on our ability to understand which parts of digital behavior interventions contribute to outcomes [17].

For DHIs to reach their full potential, we need to better understand how they are developed and what the core elements of effective interventions are [13]. This paper aims to describe the development of a multicomponent, complex DHI called “INTERCEPT” to improve secondary prevention in patients with CHD. Developed by the Irish National Institute for Prevention and Cardiovascular Health, INTERCEPT aims to promote self-management and support patients to achieve a healthy lifestyle, manage CVD risk factors, and improve adherence to cardioprotective medications. It includes an mHealth app, which integrates with a web-based health care professional (HCP) portal, a fitness wearable, and a blood pressure monitor. Responding to the need for early initiation of prevention after an index event [1], particularly given delays in patients accessing traditional (CR) programs [18,19], INTERCEPT is designed to be introduced to the patient at the time of their acute hospitalization and before discharge home. In this way, it provides a bridge to CR or an alternative for patients who choose not to join a CR program.

To maximize the potential effectiveness of INTERCEPT, the aim of this study was to adopt a theory- and evidence-based approach to development, integrating end users’ (patients and HCPs) perspectives throughout. We use the term “development” to capture the whole process from initial planning to designing and usability testing.

## Methods

### Overview

**Figure 1:**
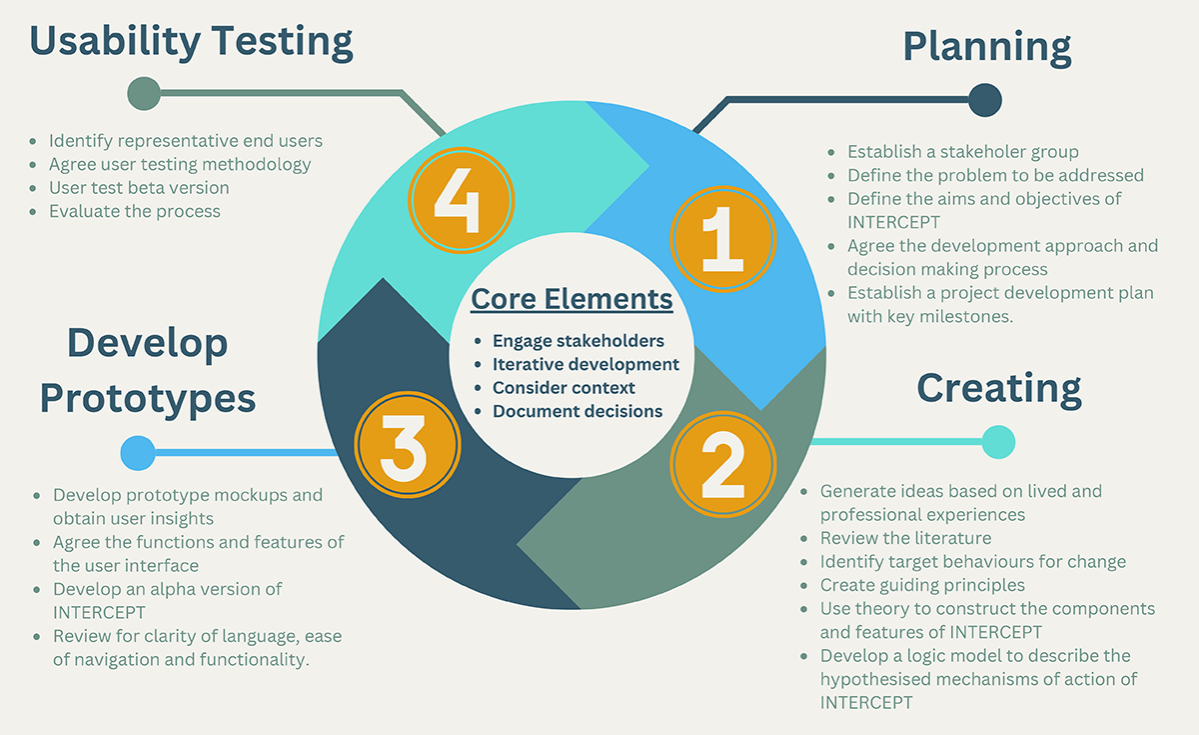
Four phases of INTERCEPT development.

We used a theory-driven, person-based approach to the development of INTERCEPT in an iterative co-design process. Our overall approach was guided by the UK Medical Research Council (MRC) framework for developing and evaluating complex interventions [20]. While co-design refers to meaningful end-user engagement across all aspects of intervention development [14], the person-based approach focuses on the psychosocial context of users and the behavioral elements of an intervention [21]. Through a series of qualitative workshops, conducted between May 2021 and December 2022, we adopted a 4-phase approach to development (Figure 1). As development commenced during the COVID-19 pandemic, all workshops were conducted online (Zoom, Zoom Video Communications). This paper is reported in accordance with the GUIDED (Guidance for Reporting Intervention Development Studies in Health Research) checklist (see Multimedia Appendix 1) [22].

### Phase 1: Planning

To ensure a diverse and inclusive approach to intervention development, an interdisciplinary stakeholder team was established. This comprised HCPs, software developers, and members from the public and patient involvement (PPI) panel of Croí (Gaelic for heart)—an Irish heart and stroke patient organization. Membership was determined by the individual’s relevant expertise in the secondary prevention of CVD, including the design of complex DHIs; health behavior change; software development; and lived experience of CVD. To establish meaningful partnership relationships and enhance engagement, roles and responsibilities were agreed upon from the outset and a process for documenting and making decisions was established [15]. Furthermore, to enable stakeholders to participate to the best of their capabilities, a flexible and supportive approach to development was adopted. For example, workshops were centered around participant availability, and supports to address digital health literacy challenges were made available. Initial meetings focused on project conceptualization and identifying the key needs and challenges to be addressed. This led to the development of the intervention aim and objectives and identification of specific target behaviors for change. These behaviors were related to lifestyle (smoking cessation, making healthy food choices, and increasing physical activity), CVD risk factor management (blood pressure, lipids, and glucose), and adherence to cardioprotective medications. A project plan with key milestones for developing INTERCEPT was established.

### Phase 2: Creating

This phase aimed to identify the guiding principles, including the design objectives, content, and features of INTERCEPT. In keeping with the person-based approach, guiding principles help to keep development focused on what is appealing and useful to the intended user, thus helping to maximize its acceptability and effectiveness [21]. INTERCEPT guiding principles were developed by (1) synthesizing the literature [10,23-26], (2) drawing on the stakeholders’ clinical and research experiences of developing and implementing DHIs [26-28], and (3) obtaining patient lived experience insights. Informed by these principles, we used social cognitive theory (SCT) [29] and select behavior change techniques (BCTs) from the behavior change taxonomy [30] to help construct the intervention components and features required to achieve the objectives of INTERCEPT. Incorporating behavior change theory in the development of health interventions is known to enhance their effectiveness [20], and SCT is one of the most commonly used theoretical frameworks in secondary prevention DHI studies [12]. This process was facilitated by a series of structured brainstorming workshops with the stakeholder team, where creative idea generation around strategies to address specific target behaviors was encouraged. Contextual insights into the clinical care pathway after a cardiac event were incorporated by involving HCPs and patients in the user journey mapping process. In line with UK MRC guidance, a logic model was developed to articulate the key components of INTERCEPT and its expected mechanisms of action [20].

### Phase 3: Developing Prototypes

To help visualize the basic layout and functionality of the app, the software developers initially produced a series of prototype mock-ups of the INTERCEPT digital interface. Through a series of workshops, these mock-ups underwent several iterations, integrating feedback from the entire stakeholder team, until a more refined design solution was reached. This led to the development of a usable first version of the app (alpha prototype), which was pilot-tested among the stakeholder team and reviewed multiple times for clarity of language, ease of navigation, and functionality. At this stage, decisions regarding the INTERCEPT logic (eg, to trigger push notifications), functional requirements (eg, registration process, user login, and safe data storage), integration of data with the HCP portal, and the use of analytics to capture usage patterns were made. Furthermore, to promote user trust and to ensure compliance with general data protection regulations (GDPR), a series of data privacy and security measures were considered in this phase. These included the introduction of encryption and access controls, adhering to data protection standards for data hosting and storage and ensuring data minimization, by developing the app to collect only the necessary personal information. In addition, a privacy policy detailing how personal data are collected, processed, and stored was developed.

INTERCEPT was developed for both iOS (Apple) and Android (Google) mobile phone platforms and was tested multiple times across a range of devices and operating systems to guarantee reliable performance and compatibility. Feedback from the workshops was collated and formulated into design specifications using a custom-designed data extraction spreadsheet. All new design features and content changes were prioritized based on their alignment to the guiding principles and their overall potential to enhance the acceptability and usability of INTERCEPT.

### Phase 4: Usability Testing

To further develop INTERCEPT, the finished product (beta version) was subjected to usability testing by a separate group of patients, who were not part of the PPI panel. Usability testing is critical to determining if an intervention is meeting the end-user needs, and, ideally, it should be completed with end users in real-life contexts [31].

### End-User Recruitment

Using purposive sampling, participants were recruited through 3 community groups, including Croí, the heart and stroke patient organization; a group representing Travellers (Indigenous ethnic minority individuals); and a farming organization. There was a specific focus on recruiting women, those living in rural isolation, and ethnic minority groups as these individuals are often underrepresented in digital health research [6,12]. Eligibility criteria included participants aged ≥40 years with a recent (≤2 years) diagnosis of CHD. Participants were required to have a smartphone, and family members were included as they play an important role in supporting engagement with technology in the home [32]. To minimize barriers to online engagement, participants were offered training in the use of Zoom technology. Furthermore, as trust in data security and privacy is a frequently reported barrier to DHI engagement [10], participants were informed of the measures taken to ensure that INTERCEPT was compliant with GDPR. We aimed to recruit a sample size of 10-12 participants to test usability, as this has been demonstrated to detect a minimum of 80% of usability problems, which is considered satisfactory for complex intervention testing [33].

### Ethical Considerations

Ethical approval for phase 4 was granted by the clinical research ethics committee at Galway University Hospitals (Ref: C.A.2797) on May 11, 2022. Informed consent was obtained from all participants, who were informed of their right to withdraw from the study at any time. Data obtained from participants was handled according to GDPR and all data were anonymous to the study team.

### Iterative Co-Design Workshops

Once consent was obtained, participants in phase 4 were provided with a link with instructions to download the app via email, a pedometer (Sportline 340; HRM USA Inc), a blood pressure monitor (UA 651 device; A&D), and a user support manual. The manual incorporated a diary, which participants were encouraged to use to document their experiences of using INTERCEPT [21]. Participants were invited to 5 online workshops over a 12-week period. This was considered sufficient time to allow participants to test the behavioral change elements of the app, such as goal setting and self-monitoring. Furthermore, this allowed for iterative amendments to be made to the app based on usability feedback. We were unable to find guidance for conducting co-design in an online environment; therefore, we adapted best practice principles for facilitation of in-person, co-design workshops, to help optimize engagement [34,35]. This involved presenting the value proposition “sharing your insights will benefit others,” having clear workshop objectives with defined roles and expectations, allowing time for participants to share their lived experiences, being supportive around the use of technology, keeping the duration of usability workshops to no longer than 1.5 hours, and offering options for out-of-hours workshops as well as individual sessions. The topic guide (see Multimedia Appendix 2) for the usability workshops was developed with input from the stakeholder team, and participants were encouraged to refer to their diary to aid recollection of their experiences.

The validated System Usability Scale (SUS) was selected to evaluate the impact of usability testing [36]. The SUS assesses components of usability, effectiveness, efficiency, and satisfaction and includes 10 questions with a Likert scale, with values ranging from 0 to 100. Participants in phase 4 were invited to complete the self-administered SUS after an initial 2-week trial of the app and after the last workshop when final modifications to the app were made. To measure eHealth literacy, the validated eHealth Literacy Scale, an 8-item scale, presented as a score out of 40, was utilized [37]. Although there is no fixed cutoff to distinguish between high and low literacy, a higher score reflects a high level of eHealth literacy. Data on baseline characteristics of participants, eHealth literacy, and the SUS were obtained by providing participants with an email link to an online survey that was created using Survey Monkey.

### Data Analysis

All quantitative data were analyzed using Stata (version 18; StataCorp). To compare the SUS data between the 2 time points, the nonparametric Mann-Whitney *U* test was used. Patient characteristics, information technology usage, and digital literacy were summarized using descriptive statistics. Qualitative data were analyzed by 2 study team members (IG and CJ) who used a deductive approach to map to 3 key aspects: usability (functionality and ease of navigation), comprehensibility (language), and content.

## Results

### Participant Characteristics

INTERCEPT followed an iterative cycle of development, involving 29 participants. These included 20 participants from the stakeholder team (4 nurse specialists, 1 physiotherapist, 1 physical activity specialist, 2 dietitians, 1 pharmacist, 2 health psychologists, 2 cardiologists, 2 software developers, and 5 PPI members), who were based in 6 different countries, and a separate group of 9 patients who were recruited for usability testing in phase 4.

### INTERCEPT Guiding Principles

A review of the associated literature identified the following key app features: self-monitoring of health behaviors, behavior change motivation, education, personalized content, ease of use, and the ability to integrate with other apps and devices [10,23]. Feedback from the stakeholder team yielded further insights. INTERCEPT should (1) engage and motivate the user, (2) facilitate psychosocial support and contact with an HCP, (3) be credible and evidence based, (4) ensure data privacy, and (5) be introduced early in the recovery journey as patients often experience delays or have limited access to CR. By consolidating findings from the literature with stakeholder team insights, we identified 5 key guiding principles for the development of INTERCEPT. An outline of these principles, comprising of design objectives and intervention features to address these objectives, are presented in Table 1.

**Table 1:** INTERCEPT design-guiding principles.

Design objectives	Intervention features
Promote user competence	Ensure simplicity across layout, language, and navigation procedures Provide clear guidance on how to use INTERCEPT
Incorporate strategies to motivate and engage users in healthy behaviors	Support goal setting, self-monitoring, and tracking of behaviors Acknowledge app usage, and provide tailored feedback on self-reported progress, using rewards where appropriate Use positive language, promoting the benefits of engaging in health behaviors
Adopt a personalized approach	Offer choice in relation to how users engage in the app (graded goal setting, turning notification on and off, timing of reminders, and personalized messages)
Promote system credibility	Be explicit in stating the organizations or people involved in developing INTERCEPT Use trusted and credible resources, with appropriate links to reputable, noncommercial organizations Comply with data privacy obligations, ensuring that appropriate data security measures are in place
Social support	Establish a communication link with health care professionals through integrating the app with a health care professional portal

### Developing Prototypes

**Figure 2:**
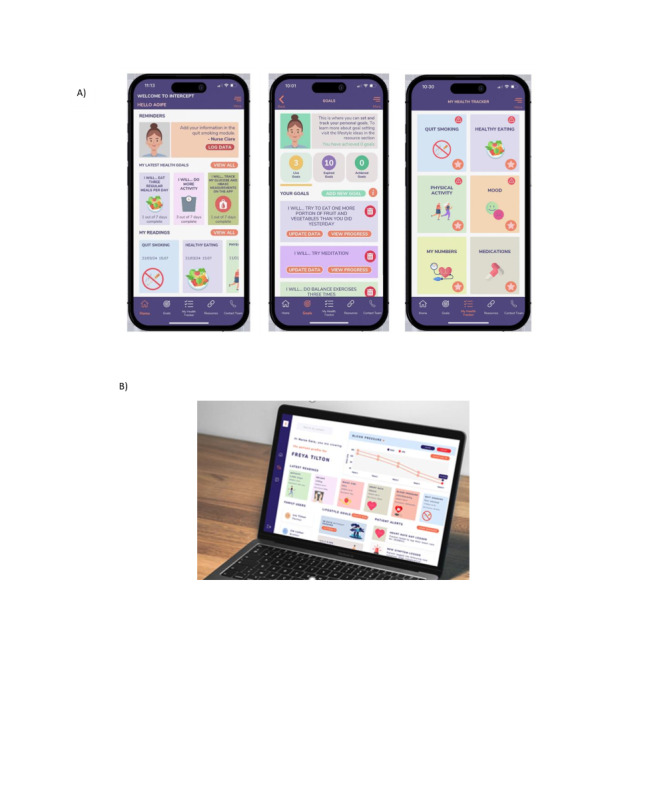
Screenshots of (A) the home screen, goal setting, and health tracker and (B) the health care professional portal.

After a series of workshops (n=7) with the stakeholder team (2 workshops with HCPs and 5 with PPI collaborators), the core components and features of INTERCEPT were agreed upon. In brief, the components included goal setting to motivate and support healthy lifestyle change; a health tracker to support self-monitoring of physical activity, mood, healthy eating, medications, blood pressure, cholesterol, and glucose levels; educational resources to increase knowledge and awareness of healthy lifestyle changes, psychosocial health, and adherence to cardioprotective medications; notifications to motivate and prompt engagement; and a link to an HCP portal to enable remote monitoring and support. A description of these components is provided in the TIDieR (Template for Intervention Description and Replication) checklist (see Multimedia Appendix 3), and screenshots are presented in Figure 2. To operationalize the intervention components and features, 14 BCTs from the taxonomy of BCTs were used. An overview of these BCTs, and how they align to the components and features of INTERCEPT, including the proposed mechanisms of actions and outcomes, is presented in the INTERCEPT logic model (see Multimedia Appendix 4). Examples of how this feedback influenced the end product features are as follows: (1) to promote user competence, additional guidance on goal setting was provided and a hard copy support manual was developed; (2) to motivate and engage users, the value proposition of the app describing its potential benefits was introduced on the home screen; (3) to ensure a personalized approach, a schedule of tailored notification messages mapped to individual usage patterns was developed; and (4) to emphasize psychological health, credible resources and messaging were included.

### Usability Testing

Usability testing was conducted between July and October 2022. In total, 11 individuals were recruited and 9 attended either the workshops (n=6) or semistructured interviews (n=3). Reasons for nonattendance were related to illness and family caring responsibilities. Baseline characteristics of participants, including demographics, CVD diagnosis, technology usage, and digital literacy, are reported in Table 2.

The mean age of participants was 62 years; 67% (6/9) were female, 89% (8/9) had a diagnosis of CVD, and 1 (11%) was a family member. Although the majority (7/9, 78%) of participants reported frequently using apps, the mean eHealth literacy score was 27.6 (SD 6.1). While acknowledging the small sample size, improvements were identified in overall mean SUS scores from 60.8 (SD 23.5) at commencement of usability testing to 82.8 (SD 7.1) after usability testing (*P*=.02; Table 3). Although improvements were observed across all 10 questions, they were significant for questions 2 (I found the app unnecessarily complex; *P*=.04), 3 (I thought the app was easy to use; *P*=.02), 5 (I found the various functions of the app well integrated; *P*=.03), and 9 (I felt confident using the app; *P*=.03).

**Table 2:** Baseline characteristics of usability testing participants.

Characteristic				Value		
**Demographics (n=9)**						
	Gender (female), n (%)		6 (67)			
	Age (y), mean (SD)		62.2 (11.4)			
	Enrolled in GMS^a^ scheme, n (%)		7 (78)			
**Geographical region (n=9), n (%)**						
	Connacht		4 (44)			
	Leinster		3 (33)			
	Munster		2 (22)			
	Urban		3 (33)			
	Rural		6 (67)			
**Ethnicity (n=9), n (%)**						
	White Irish		8 (89)			
	Irish Traveller		1 (11)			
**Education level (n=9), n (%)**						
	Primary education		1 (11)			
	Secondary education		3 (33)			
	Technical or vocational		1 (11)			
	Third level diploma or degree		4 (44)			
**Cardiovascular diagnosis (self-reported;n=8), n (%)**						
	Myocardial infarction and percutaneous coronary intervention		3 (33)			
	Heart failure		2 (22)			
	Percutaneous coronary intervention		1 (11)			
	Heart valve disease		1 (11)			
	Atrial fibrillation		1 (11)			
**Cardiac rehabilitation program completion (n=8), n (%)**				7 (78)		
	In person		4 (57)			
	Online		3 (43)			
**Information technology and digital literacy (n=8)**						
	**Access to internet at home, n (%)**		9 (100)			
	**How often do you use apps on your phone? n (%)**					
		Frequently			7 (78)	
		Occasionally			1 (11)	
		Rarely			1 (11)	
	**eHEALS^b^ score, mean (SD)**					27.6 (6.1)

^a^GMS: General Medical Scheme (refers to the means tested provision of state health care).

^b^eHEALS: eHealth Literacy Scale.

Across the 3 key areas of usability, comprehensibility, and content, participants made 64 suggestions for INTERCEPT, and of these, 51 were implemented. Through stakeholder consensus, reasons for not implementing suggestions were related to practical considerations (budget and time) and misalignment with the guiding principles. A summary of suggestions, across the themes of usability, comprehensibility, and content, were mapped to the intervention components (Multimedia Appendix 5). The following are selected insights. To enhance functionality and minimize the user burden associated with manual data entry, participants identified the importance of integrating data from the fitness wearable and blood pressure monitor with INTERCEPT. However, this change was not feasible to implement immediately, as it required consideration of multiple technical and user experience factors. These included ensuring reliable data transfer and compatibility between INTERCEPT and other devices; privacy and security; and the provision of adequate technical support, including user instructions and manuals to guide setup. Although addressing these factors required additional time and resources, data integration was achieved following usability testing. To motivate the user, participants recommended including rewards for setting and achieving goals and using language to promote more personal ownership, for example, changing “your data” to “my data.” In recognizing the acute clinical context of implementation, participants emphasized the importance of including welcoming messages on the home screen to acknowledge the early stages of recovery. To enhance navigation, participants recommended greater integration between components. For example, if a user achieves a low mood score, they should receive prompts with links to the goal setting and resource sections, rather than having to access these components separately.

**Table 3:** Pre- and postusability testing System Usability Scale scores.

Question	Preusability testing (n=9), mean (SD)	Postusability testing (n=8), mean (SD)	*P* value
1. I think that I would like to use the app frequently	2.7 (1.4)	3.5 (1.1)	.12
2. I found the app unnecessarily complex	2.6 (1.1)	3.6 (0.5)	.04
3. I thought the app was easy to use	2.6 (1.2)	3.8 (0.5)	.02
4. I think that I would need assistance to be able to use this app	2.1 (1.3)	2.6 (0.9)	.39
5. I found the various functions in the app were well integrated	2.3 (1.0)	3.4 (0.7)	.03
6. I thought there was too much inconsistency in the app	2.3 (1.1)	3.1 (0.4)	.05
7. I would imagine that most people would learn to use this app very quickly	2.3 (1.2)	3.3 (1.0)	.08
8. I found the app very cumbersome or awkward to use	2.4 (1.2)	3.4 (0.5)	.06
9. I felt very confident using the app	2.8 (0.7)	3.5 (0.5)	.03
10. I needed to learn a lot of things before I could get going with the app	2.2 (1.6)	3.0 (0.5)	.37
Total score	60.8 (23.5)	82.8 (7.1)	.02

## Discussion

### Principal Findings

This paper describes the methods used to develop INTERCEPT, a multicomponent DHI, which integrates a smartphone app, HCP portal, fitness wearable, and blood pressure monitor to improve secondary prevention in patients with CHD. By providing a comprehensive and transparent description of methods used, we envision that our reporting will facilitate replication of the design process for future DHIs while also contributing to the growing science of intervention development. For creating effective DHIs, the use of the best combination of approaches to intervention development is required [38]. Accordingly, we used a combination of behavioral change theory, co-design, and the person-based approach to developing INTERCEPT. To our knowledge, this is one of the first studies to adopt this approach for the development of mHealth secondary prevention interventions. The broad range of actions undertaken across the development phases ensured the success of this approach. Many of these actions, for example, planning, designing, and creating, are consistent with guidance from the taxonomy of approaches to developing interventions by O’Cathain et al [39]. This highlights the relevance of using a taxonomy for future CVD DHI development research.

Developing DHIs is increasingly being recognized as a transdisciplinary endeavor, and meaningful stakeholder involvement is critical to successful co-design [15,38]. Despite this, a recent scoping review revealed that establishing meaningful relationships was the least reported process used in co-design studies in CVD secondary prevention [15]. Moreover, PPI is often absent from approaches to intervention development [39]. We addressed this by engaging PPI early in the development process and by using specific strategies, such as agreeing roles and responsibilities to foster meaningful relationships. Given the high level of stakeholder diversity (HCPs, patients, and software developers), the role of facilitators (study team members IG and CJ) was critical to ensuring an authentic understanding of the stakeholders’ real-world experiences. Through their backgrounds as CVD nurse specialists, they were able to operate in an empathetic way, while also managing potential power imbalances between members of the stakeholder team. The interdisciplinary expertise of the stakeholder team helped to ensure that the users’ needs and preferences, the influence of behavioral theory, best practice CVD prevention guidelines, and technical and practical factors were considered throughout development. Through dynamic, iterative cycles of development, this resulted in the development of a complex intervention, targeting multiple behaviors for change through a personalized user interface, with bidirectional communication via an HCP portal and remote monitoring technology. Consistent with findings from other DHI co-design studies, having the same PPI and HCP group involved throughout enhanced this process, allowing for more in-depth and intensive iteration along the development continuum [35,40].

Although most secondary prevention DHIs focus on outpatient settings, INTERCEPT is designed to be introduced to patients as early as possible after the diagnosis of CHD [41]. Based on recent systematic review and realist synthesis evidence, focusing on early engagement, ensuring a personalized approach, and providing opportunities to connect with an HCP are all features associated with improved participation in telehealth CR and CVD health outcomes [42]. Although HCP support has been identified as one of the most important factors associated with engagement in mHealth DHIs [25], it is important to establish the extent to which it can add further value, without becoming resource intensive [43]. The INTERCEPT portal strives to achieve this balance through protocol-driven remote monitoring and support based on real-time data.

To our knowledge, INTERCEPT is one of the few secondary prevention mHealth DHIs that target multiple behaviors for change. This is particularly important as lifestyle- and medical-related CVD risk factors and their corresponding behaviors are strongly interlinked [1]. Although the guiding principles were key to identifying key components and features to help address these behaviors, using BCTs targeting determinants of SCT helped to operationalize them. Many of the BCTs included in INTERCEPT (eg, self-monitoring of behavior [2.3], information about health consequences [5.1], and goal setting [1.1]) align to recent systematic review evidence for effective BCTs in CR DHIs [12]. By presenting an overview of these BCTs as part of the INTERCEPT logic model, we respond to the increasing calls for explicit reporting of components and expected mechanisms of action of co-designed interventions [15,20].

Similar to findings by Tay et al [44], this paper highlights that it is feasible to develop DHIs in an online environment. Importantly, this enabled us to foster an inclusive approach to intervention development where geography was not a barrier. Indeed, the diverse group of representative end users in the stakeholder team and recruited for usability testing was a key strength of the INTERCEPT development process. For example, among those recruited for usability testing, one-third had never previously attended CR; almost 70% were women; 78% were enrolled in the General Medical Scheme (means tested provision of state health care); 1 individual identified as an Irish Traveller; and eHealth literacy levels were mixed, with scores ranging from 15 to 34 out of 40 (mean score 27.6). Given how cultural and socioeconomic factors including gender and digital literacy influence the acceptability of DHIs, involving diverse populations in their design is essential to optimizing their potential benefits [5]. Outcomes from the usability-testing phase resulted in a number of refinements being made to INTERCEPT. Data from the SUS evaluation suggest that these refinements potentially led to improvements in its usability. This highlights the important value of usability testing in optimizing the user experience and overall quality of the product [35].

Following UK MRC guidance for developing and evaluating complex interventions, our next step in the intervention development process is to evaluate the feasibility of INTERCEPT among a sample of patients with CHD in a real-world clinical setting. By conducting a nonrandomized, mixed methods, feasibility study, the acceptability and usability of INTERCEPT will be assessed. These insights will help to (1) inform further refinement of INTERCEPT and (2) determine the feasibility of a definitive randomized controlled trial. The protocol for this feasibility study is detailed elsewhere [45].

### Limitations

The INTERCEPT development process had some limitations. First, the outcomes of our co-design process focus primarily on the development of INTERCEPT. However, there is also a need to report on stakeholder experiences and cost-effectiveness [14,46]. This would help address the paucity of data on the impact of co-design processes on stakeholders, while also informing strategies to optimize participatory approaches for intervention development [47]. Second, co-design is time-consuming, and the evolving nature of technology demands more fast-paced design processes to ensure that DHIs remain relevant and engaging [46]. Although our development timeframe was consistent with in person co-design studies [13], further research is required to ascertain if online approaches can improve efficiencies. Third, usability testing of INTERCEPT required participants to retrospectively report their experiences over a defined period of time, which may have resulted in insights been forgotten or distorted. Including the use of think aloud interviews, whereby participants are asked to give their immediate reactions to every element, may help to address this. However, further guidance on using this approach online is required. Fourth, social desirability bias may have influenced the SUS results; to address this, the survey was self-administered and the results were anonymous to the study team. Finally, because of the low sample size, the SUS results should be interpreted with caution; further research with a larger sample size is warranted to help evaluate the usability testing process.

### Conclusions

The proliferation of DHIs including mHealth has enabled the development of new and innovative approaches for the secondary prevention of CVD. However, careful attention to the development of these DHIs is required to increase their effectiveness and uptake in clinical practice. This paper illustrates how combining behavior change theory with a person-based, co-design approach to DHI development is feasible and can result in the successful development of an intervention that responds to end users’ needs and preferences, including desired content and features. Additionally, our comprehensive reporting offers guidance to other researchers for developing future DHIs, thus facilitating the translation of evidence into practice.

## Appendix

Multimedia Appendix 1 GUIDED (Guidance for Reporting Intervention Development Studies in Health Research) checklist.

Multimedia Appendix 2 Topic guide for usability testing.

Multimedia Appendix 3 TIDieR (Template for Intervention Description and Replication) checklist.

Multimedia Appendix 4 INTERCEPT logic model.

Multimedia Appendix 5 Insights from usability testing.

## Abbreviations

BCT: behavior change technique
CHD: coronary heart disease
CR: cardiac rehabilitation
CVD: cardiovascular disease
DHI: digital health interventions
GDPR: general data protection regulations
GUIDED: Guidance for Reporting Intervention Development Studies in Health Research
HCP: health care professional
mHealth: mobile health
MRC: Medical Research Council
PPI: public and patient involvement
SCT: social cognitive theory
SUS: System Usability Scale
TIDieR: Template for Intervention Description and Replication
