# Supplementary material for: Development of a Digital Health Intervention for the Secondary Prevention of Cardiovascular Disease (INTERCEPT): Co-Design and Usability Testing Study
Source: JMIR Hum Factors. 2024 Oct 23;11:e63707. doi: 10.2196/63707 (PMC11541151; doi:10.2196/63707)
Supplement: Multimedia Appendix 2 [file humanfactors_v11i1e63707_app2.docx]

**Multimedia Appendix 2: Topic Guide for Usability Testing Workshops**

| **Components of the INTERCEPT App** | **Comments:** |
| --- | --- |
| Were you able to find the instructions on how to use the app?  How helpful were these instructions? |  |
| **Home Screen**  Was it easy to understand the home screen? |  |
| **Goal setting section**   - How easy was it to understand the goal setting section? - Was the importance of goal setting clear to you? (If no please comment) - Were you able to set yourself goals? (If no please comment) - Were you able to track your goals? (If no please comment) - How motivational did you find this section? - What did you most like about this section? - What did you least like about this section |  |
| **My Health Tracker:**   - How easy was it to understand the health tracker section? - Was the importance of this section clear to you? (if no please comment) - How easy or difficult was it to log your information across all sections including mood, physical activity, cholesterol, blood pressure, cholesterol and weight? (what might make it easier?) - What did you most like about this section? - What did you least like about this section? |  |
| **Resources**   - Was the importance of this section clear to you? (If no please comment) - Was there sufficient information provided in this section? - What did you like most about this section? - What did you like least about this section? |  |
| **General Comments**  Did the app maintain your interest and attention? (if no please comment)  What did you think of the look and feel of the app?  What aspects of the app did you like the most?  How easy or difficult was it to move between screens in the app?  Did you receive notifications from the app?  Were these notifications helpful or unhelpful?  Have you any suggestions that might help improve the app? |  |
