# Supplementary material for: Development of a Digital Health Intervention for the Secondary Prevention of Cardiovascular Disease (INTERCEPT): Co-Design and Usability Testing Study
Source: JMIR Hum Factors. 2024 Oct 23;11:e63707. doi: 10.2196/63707 (PMC11541151; doi:10.2196/63707)
Supplement: Multimedia Appendix 3 [file humanfactors_v11i1e63707_app3.docx]

**
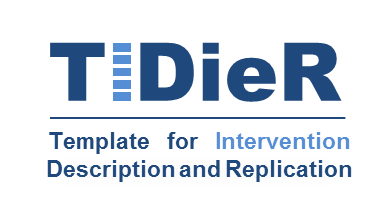
The TIDieR (Template for Intervention Description and Replication) Checklist*:**

Information to include when describing an intervention and the location of the information

| **Item number** | **Item** | **Where located **** | |
| --- | --- | --- | --- |
|  |  | Primary paper  (page or appendix  number) | Other ^†^ (details) |
|  | **BRIEF NAME** |  |  |
| **1.** | Provide the name or a phrase that describes the intervention.  *INTERCEPT is a digital health intervention (DHI) for the secondary prevention of cardiovascular disease (CVD)* |  | ______________ |
|  | **WHY** |  |  |
| **2.** | Describe any rationale, theory, or goal of the elements essential to the intervention  *The main objective of INTERCEPT is to promote self-management and support patients to achieve a healthy lifestyle, manage CVD risk factors, and improve adherence with cardio-protective medications. The development of INTERCEPT has been informed by social cognitive theory and select behaviour change techniques from the taxonomy of behaviour change techniques (BCTs). These are illustrated in figure1. Responding to the need for early initiation of prevention following an index event, INTERCEPT is designed to be introduced to the patient at the time of their acute hospitalisation and before discharge home.* |  | _____________ |
|  | **WHAT** |  |  |
| **3.** | Materials: Describe any physical or informational materials used in the intervention, including those provided to participants or used in intervention delivery or in training of intervention providers.  *INTERCEPT includes a mHealth app, which integrates with a web-based health care professional (HCP) portal, a fitness wearable and blood pressure monitor. Of these digital components, patients are provided with the mHealth app, a fitness wearable and a blood pressure monitor. In addition, they receive a hard copy user support manual and are provided with a link to access frequently asked questions (FAQs).* |  | _____________ |
| **4.** | *Procedures: Describe each of the procedures, activities, and/or processes used in the intervention, including any enabling or support activities.*  *The components of INTERCEPT include: a* ***home screen*** *to support app navigation; tailored* ***goal setting*** *to motivate and support healthy lifestyle changes; a* ***health tracker*** *to support self-monitoring of physical activity, mood, healthy eating, medications, blood pressure, cholesterol and glucose levels; educational* ***resources*** *to increase knowledge and awareness of healthy lifestyle changes and adherence with cardio-protective medications and* ***notifications*** *to prompt engagement with the INTERCEPT.*  ***Home Screen***  *This is the first screen that users see when they open the app. It includes a personalised welcome from Nurse Ciara, with specific pop up messages, including instructions for new users. It presents a quick reference overview of goal progress and key readings for example, blood pressure which the user can favourite for viewing. The link at the top of the screen "more" provides access to the privacy policy, module settings and instructions. The menu bar along the bottom gives users easy access to the app modules, which include goal setting, my health tracker, resources and contact team.*  ***Goal Setting***  *This page allows the user to set individual goals across the categories of healthy eating, physical activity, stress management, blood pressure, weight managing, quit smoking, cholesterol, HbA1c and blood glucose and mood. Users can choose to select from a menu of pre-set goals or can create their own personalised goals. To set a goal, users must specify the duration and frequency of the goal. They can track their progress, which is visually represented using a graph and a dashboard, which shows the number of active goals, achieved goals and expired goals. Users receive notifications and motivational messages to prompt them to both set and track their goals. They receive a reward (trophies) when they both set and achieve goals.*  ***Health tracker***  *This page is designed to support self-monitoring of progress across physical activity, mood, healthy eating, smoking, medications, weight, blood pressure, cholesterol, HbA1c and glucose levels. Users can integrate data from their blood pressure monitor and fitness wearable in this section. Tailoring of messages, with recommended actions, including follow-up at general practice are based on responses to pre-set questions and data entered. Feedback is presented visually through graphs and progress can be viewed daily, weekly and monthly. When the user enters their data they receive motivational messages to provide positive reinforcement when progressing well towards their goals or as messages of encouragement if progress is not as expected*  ***Resources***  *Provision of education information using interactive, evidence-based, credible resources from Irish organisations such as Croí (the heart and stroke patient organisation) and the Health Service Executive (HSE). These include: Lifestyle Ideas (goals setting, keeping active, eating to protect my heart, moving towards a healthy weight and shape); My Mind Health (keeping stress levels down, my mood & useful websites); Protective Medications for your Heart; Knowing My Numbers (blood pressure, cholesterol and blood glucose and diabetes); Your Heart (Heart Conditions explained and support services) and training on how to measure blood pressure. Users have the option to favourite resources that are most relevant to them.*  ***Notifications***  *The INTERCEPT uses push notifications to engage users who are not using the app. In-app notifications are designed to prompt the users to self-monitor their goals, track physiological and lifestyle parameters and to take their medications. Users have the option of turning the in-app notifications on or off. Notifications are designed to be positive and to give some interesting information about the app before inviting them to use it.*  *Examples of push notifications include:*   - *We've missed you, check the INTERCEPT app to let us know how you are getting on* - *All physical activity counts! Let us know how you're doing.* - *When it comes to recovering from a heart event, looking after your emotional health is just as important as your physical health.*   ***Health care professional (HCP) portal***  *The portal provides a structured and functional platform for the HCP to monitor patient health data and engagement with INTERCEPT. The HCP can view data submitted by patients via the app. These data include: physiological data (weight, BMI, LDL cholesterol, blood pressure, heart rate, glucose and HbA1c); medication usage; goals set and responses to pre-set questions regarding mood, physical activity and diet. Custom algorithms are used to organise various types of data into a single, coherent table, presenting a clear picture of each patient's progress on a dashboard. The system is designed to identify and highlight unusual or concerning health data to ensure prompt attention from the HCP. Based on this data the HCP will communicate with the user and will make recommendations for follow-up with general practice if required. These communications will be made via telephone or email based on the users preference.* |  | _____________ |
|  | **WHO PROVIDED** |  |  |
| **5.** | For each category of intervention provider (e.g. psychologist, nursing assistant), describe their expertise, background and any specific training given.  *The INTERCEPT delivery team will comprise of cardiovascular nurse specialists who will be trained in the use of INTERCEPT, including technology deployment, and monitoring of the health care professional portal. These nurses must have relevant clinical experience in the secondary prevention of CVD. To supplement training, the nurses will be provided with 2 manuals to support: 1) the on boarding of patients to INTERCEPT and 2) the use of the HCP portal*. |  | _____________ |
|  | **HOW** |  |  |
| **6.** | Describe the modes of delivery (e.g. face-to-face or by some other mechanism, such as internet or telephone) of the intervention and whether it was provided individually or in a group.  *INTERCEPT is a DHI that is designed to be delivered using a mobile health app with integration with a fitness wearable and blood pressure monitor. Patients will received 1:1 training in the use of INTERCEPT prior to their discharge from hospital*. |  | _____________ |
|  | **WHERE** |  |  |
| **7.** | Describe the type(s) of location(s) where the intervention occurred, including any necessary infrastructure or relevant features.  *For the feasibility study, patients will be introduced to INTERCEPT in the acute clinical setting of the Cardiology and Cardiothoracic Departments of University Hospital Galway, Ireland.* |  | _____________ |
|  | **WHEN and HOW MUCH** |  |  |
| **8.** | Describe the number of times the intervention was delivered and over what period of time including the number of sessions, their schedule, and their duration, intensity or dose.  *In line with the nature of DHIs, INTERCEPT is readily available for patients to use in accordance with their preferences in their own home. Usage patterns will be monitored as part of the feasibility study.* |  | _____________ |
|  | **TAILORING** |  |  |
| **9.** | If the intervention was planned to be personalised, titrated or adapted, then describe what, why, when, and how.  *Adopting a personalised approach is a key guiding principle of INTERCEPT. Users are offered choice in how they engage with the app. For example, they can turn notifications on/off, they can set reminders to take their medications and they can either create or select goals from a drop down list of examples. All notification are personalised and a schedule of tailored notification messages mapped to individual usage patterns has been developed.* |  | _____________ |
|  | **MODIFICATIONS** |  |  |
| **10.^ǂ^** | If the intervention was modified during the course of the study, describe the changes (what, why, when, and how).  *N/A - a pilot feasibility study of INTERCEPT is currently in progress and outcomes are awaited* |  | _____________ |
|  | **HOW WELL** |  |  |
| **11.** | Planned: If intervention adherence or fidelity was assessed, describe how and by whom, and if any strategies were used to maintain or improve fidelity, describe them.  *While assessing fidelity is beyond the scope of the feasibility study, this will be assessed in a future RCT* |  | _____________ |
| **12.^ǂ^** | Actual: If intervention adherence or fidelity was assessed, describe the extent to which the intervention was delivered as planned.  *N/A* |  | _____________ |

** **Authors** - use N/A if an item is not applicable for the intervention being described. **Reviewers** – use ‘?’ if information about the element is not reported/not sufficiently reported.

† If the information is not provided in the primary paper, give details of where this information is available. This may include locations such as a published protocol or other published papers (provide citation details) or a website (provide the URL).

ǂ If completing the TIDieR checklist for a protocol, these items are not relevant to the protocol and cannot be described until the study is complete.

* We strongly recommend using this checklist in conjunction with the TIDieR guide (see *BMJ* 2014;348:g1687) which contains an explanation and elaboration for each item.

* The focus of TIDieR is on reporting details of the intervention elements (and where relevant, comparison elements) of a study. Other elements and methodological features of studies are covered by other reporting statements and checklists and have not been duplicated as part of the TIDieR checklist. When a **randomised trial** is being reported, the TIDieR checklist should be used in conjunction with the CONSORT statement (see [www.consort-statement.org](http://www.consort-statement.org)) as an extension of **Item 5 of the CONSORT 2010 Statement.** When a **clinical trial** **protocol** is being reported, the TIDieR checklist should be used in conjunction with the SPIRIT statement as an extension of **Item 11 of the SPIRIT 2013 Statement** (see [www.spirit-statement.org](http://www.spirit-statement.org)). For alternate study designs, TIDieR can be used in conjunction with the appropriate checklist for that study design (see [www.equator-network.org](http://www.equator-network.org)).
