# Supplementary material for: Development of a Digital Health Intervention for the Secondary Prevention of Cardiovascular Disease (INTERCEPT): Co-Design and Usability Testing Study
Source: JMIR Hum Factors. 2024 Oct 23;11:e63707. doi: 10.2196/63707 (PMC11541151; doi:10.2196/63707)
Supplement: Multimedia Appendix 4 [file humanfactors_v11i1e63707_app4.pdf]

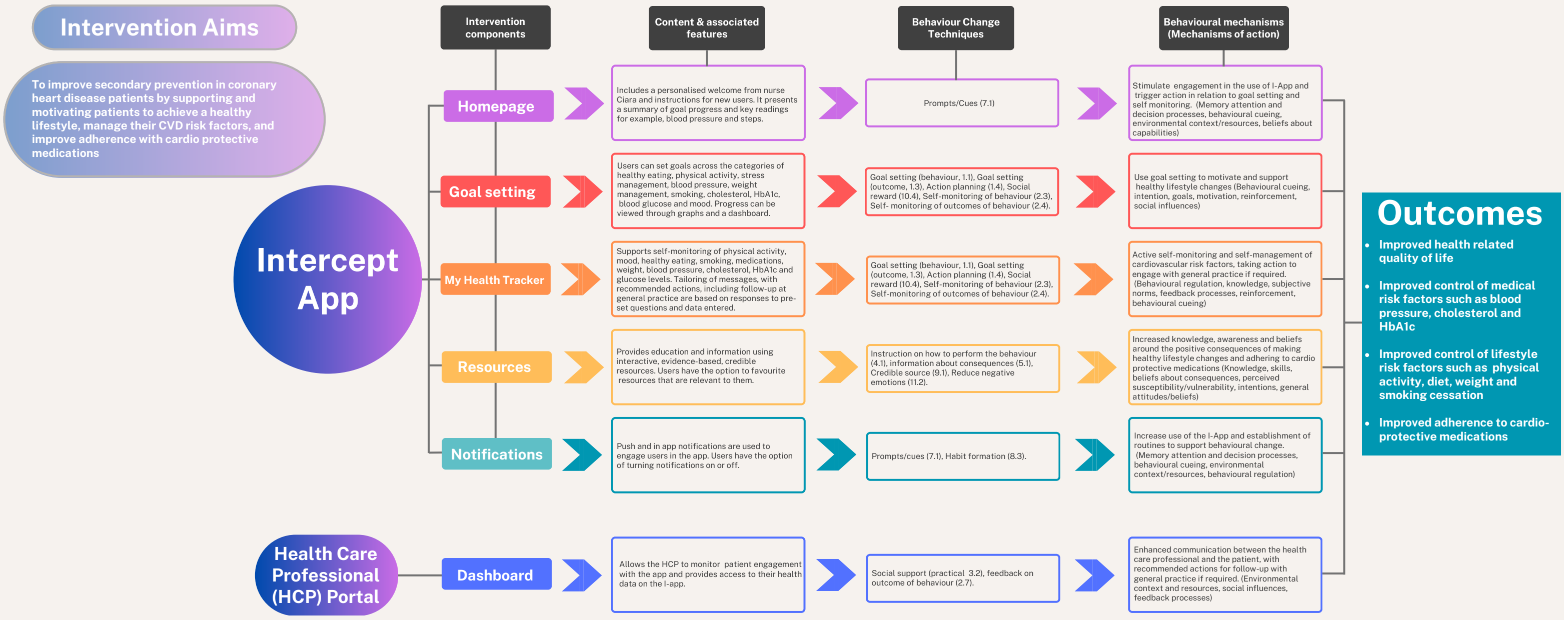

Intervention Aims
To improve secondary prevention in coronary heart disease patients by supporting and motivating patients to achieve a healthy lifestyle, manage their CVD risk factors, and improve adherence with cardio protective medications
Intercept App
Health Care Professional (HCP) Portal
Intervention components
Content & associated features
Behaviour Change Techniques
Behavioural mechanisms (Mechanisms of action)
Homepage
Includes a personalised welcome from nurse Ciara and instructions for new users. It presents a summary of goal progress and key readings for example, blood pressure and steps.
Prompts/Cues (7.1)
Stimulate engagement in the use of I-App and trigger action in relation to goal setting and self monitoring. (Memory attention and decision processes, behavioural cueing, environmental context/resources, beliefs about capabilities)
Goal setting
Users can set goals across the categories of healthy eating, physical activity, stress management, blood pressure, weight management, smoking, cholesterol, HbA1c, blood glucose and mood. Progress can be viewed through graphs and a dashboard.
Goal setting (behaviour, 1.1), Goal setting (outcome, 1.3), Action planning (1.4), Social reward (10.4), Self-monitoring of behaviour (2.3), Self- monitoring of outcomes of behaviour (2.4).
Use goal setting to motivate and support healthy lifestyle changes (Behavioural cueing, intention, goals, motivation, reinforcement, social influences)
My Health Tracker
Supports self-monitoring of physical activity, mood, healthy eating, smoking, medications, weight, blood pressure, cholesterol, HbA1c and glucose levels. Tailoring of messages, with recommended actions, including follow-up at general practice are based on responses to pre-set questions and data entered.
Goal setting (behaviour, 1.1), Goal setting (outcome, 1.3), Action planning (1.4), Social reward (10.4), Self-monitoring of behaviour (2.3), Self-monitoring of outcomes of behaviour (2.4).
Active self-monitoring and self-management of cardiovascular risk factors, taking action to engage with general practice if required. (Behavioural regulation, knowledge, subjective norms, feedback processes, reinforcement, behavioural cueing)
Resources
Provides education and information using interactive, evidence-based, credible resources. Users have the option to favourite resources that are relevant to them.
Instruction on how to perform the behaviour (4.1), information about consequences (5.1), Credible source (9.1), Reduce negative emotions (11.2).
Increased knowledge, awareness and beliefs around the positive consequences of making healthy lifestyle changes and adhering to cardio protective medications (Knowledge, skills, beliefs about consequences, perceived susceptibility/vulnerability, intentions, general attitudes/beliefs)
Notifications
Push and in app notifications are used to engage users in the app. Users have the option of turning notifications on or off.
Prompts/cues (7.1), Habit formation (8.3).
Increase use of the I-App and establishment of routines to support behavioural change. (Memory attention and decision processes, behavioural cueing, environmental context/resources, behavioural regulation)
Dashboard
Allows the HCP to monitor patient engagement with the app and provides access to their health data on the I-app.
Social support (practical 3.2), feedback on outcome of behaviour (2.7).
Enhanced communication between the health care professional and the patient, with recommended actions for follow-up with general practice if required. (Environmental context and resources, social influences, feedback processes)
Outcomes
• Improved health related quality of life
• Improved control of medical risk factors such as blood pressure, cholesterol and HbA1c
• Improved control of lifestyle risk factors such as physical activity, diet, weight and smoking cessation
• Improved adherence to cardio-protective medications
