# Supplementary material for: Development of a Digital Health Intervention for the Secondary Prevention of Cardiovascular Disease (INTERCEPT): Co-Design and Usability Testing Study
Source: JMIR Hum Factors. 2024 Oct 23;11:e63707. doi: 10.2196/63707 (PMC11541151; doi:10.2196/63707)
Supplement: Multimedia Appendix 5 [file humanfactors_v11i1e63707_app5.docx]

**Multimedia Appendix 5. Selected insights from usability testing with resulting actions**

| **Intercept component** | **Identified issue** | **Quote** | **Action** |
| --- | --- | --- | --- |
| **Home page** |  |  |  |
| Usability (functionality & navigation) | 1)Instructions on how to use the app could appear more frequently at the beginning of use  2)The home screen looks very busy, could it be streamlined by removing goals setting | *“The initial look and feel is too busy, does goal setting need to be included, I don’t know what this means it could be off putting especially if you are trying to come to terms with your diagnosis”* | 1)A pop up instruction for each page of the app was developed, with the option to turn off if required  2)Rather than removing goal setting from the home screen, greater instructions on how to set goals through tips were added |
|  |  |  |  |
| Content (text and visuals) | 1)Home screen would benefit from some gentle welcoming messages  2)Could Nurse be Ciara be more real and avator like | *“For someone who has found out that they have a heart problem heart the I-App might seem daunting and overwhelming so having some sort of a welcome message would help”* | 1)A new messaging sequence to welcome new users was added. For example  “Welcome to the Intercept app and to the start of your recovery journey. Recovering from a heart event can take time, so pace yourself and get to know the app in your own time”  2) Developing an avator is beyond the current scope and budget of the project. |
| **Goal Setting** |  |  |  |
| Usability (functionality & navigation) | 1. Use reminders to prompt you to set goals and to check in on your progress in terms of achieving goals 2. Put a limit on the amount of goals one can set 3. Can the goals set by patients be available for viewing on the portal 4. The ability to see progress could be enhanced by having a line joining the dots on the graphs 5. Similar to goal setting, rewards for goal attainment should be used 6. To help set goals it would be useful if this section of the app could link to the resource section on how to set goals 7. Can a list of achieved goals be displayed as this will help build confidence and self-efficacy | *“People could get carried away and set too many goal and then not achieve any, which could turn them off using the app”* | 1. Notification were added to prompt goal setting and to update progress 2. While a limit was not added a new hint to the goal setting tips was added “Be realistic with your goals and dont set too many on one go" 3. A new function visualising goals was included on the nurse portal. 4. Graphs were updated to display this connection 5. Rewards for goal attainment we added 6. Additional links were added to facilitate this 7. An additional function was added to capture achieved goals. |
| Comprehensibility (Language) | Text "edit data" is unclear, can this be rephrased to "update your data" |  | This revision to text was made |
| Content (text and visuals) | 1) There was confusion around the meaning of goal types and the use of yes/no responses  2) Nurse Ciara hints on goal setting don't stand out enough, writing is too small, perhaps bold the orange font against the grey | “I really don’t understand what is meant by goal types” | 1)This was removed from the app  2)Font size was increase and colours were adjusted. |
| **My Health tracker** |  |  |  |
| Usability (functionality & navigation) | 1. Beyond steps it would be good to capture active minutes from other activities such as swimming and yoga 2. Steps should automatically integrate from your phone or fitness wearable to the I-App 3. Rewards for tracking physical activity, medications usage and blood pressure would be beneficial 4. As the my numbers section is very popular, users requested the ability to favourite certain numbers, for example blood pressure 5. Can the time of BP reading be captured as date will not help differentiate between am/pm readings 6. Can instructions on how to bypass the duration of prescriptions be included 7. If the person is consistently scoring low on mood or has high blood pressure or if they are not taking their medications can this be flagged on the health care portal 8. There needs to be greater linkage between the components in this section and the goal setting and resource sections | *“I don’t see the benefit of using the physical activity section unless it can automatically link to my fitness watch, all devices should talk to each other”* | 1. Through future integration of fitness wearables this will be achieved 2. This will be achieved as part of the above action 3. This is beyond scope as it would require substantial design and development work 4. The favourite function was extended to the my numbers section. 5. This feature was added 6. More simplified instructions were introduced 7. Alerts for concerning health data were introduced to the healthcare portal 8. Additional links were introduced to ensure more seamless and intuitive navigation between section |
| Comprehensibility | 1)Change the words “your data” to “my data”  2)Change the word “access” to “get to” |  | Both of these updates were made |
| Content | 1)Can a more comprehensive list of medications be included under the categories  2)Can an information icon with additional information on LDL, Glucose and HbA1c be introduced, similar to healthy eating and physical activity  3)The presentation of the blood pressure data could be improved to represent daily, weekly and monthly readings  4)Additional guidance on how frequently BP and LDL should be checked was requested  5) Introduce tips for when people score low on their mood |  | 1)The list was updated across all medication categories.  2) Additional content was developed to facilitate this update  3) This update was made  4) Additional content was introduced to provide this guidance  5) 5 tips were developed for low mood scores example “Talk to someone about what is bothering you” |
| **Resources** | | | |
| Usability (functionality & navigation) | 1. Really like the videos, placing them before any other resource materials would be more engaging 2. Minimise the use of web links as they don’t always work. | *“There is so much useful information in this section, however its far more engaging to watch videos than read text”* | 1. The sequencing of the materials in the resource section was re-ordered 2. Broken links were removed and PDFs were included where available for resources |
| Content (text and visuals) | 1. Include a testimonial from patient talking about their experiences of recovering from heart disease 2. More practical information on travel and health insurance would be helpful to include 3. Change the infographic to one that convey resources/information | *“Its always good to feel like you are not alone and hearing from other patients can help, even if it is just a short video”* | 1. This is beyond scope but could be considered in a future version, with additional funding 2. This will be considered for future versions 3. An new infographic for this section was designed. |
